# Severe Maternal Morbidity: The Impact of Race on Tricare Beneficiaries

**DOI:** 10.7759/cureus.68620

**Published:** 2024-09-04

**Authors:** Eleanor P Iodice, Rachel Tindal, Katherine R Porter, Emily Lyon, Amanda Hall, Veronica M Gonzalez-Brown, Erin A Keyser

**Affiliations:** 1 Obstetrics and Gynecology, Lincoln Memorial University DeBusk College of Osteopathic Medicine, Knoxville, USA; 2 Obstetrics and Gynecology, San Antonio Uniformed Services Health Education Consortium, Ft. Sam Houston, USA; 3 Obstetrics and Gynecology, Mike O'Callaghan Military Medical Center, Nellis Air Force Base, USA; 4 Obstetrics and Gynecology, Wright-Patterson Medical Center, Wright-Patterson Air Force Base, USA

**Keywords:** healthcare disparities, maternal morbidity-maternal death, severe maternal morbidity, insurance, health care disparities, obstetrics

## Abstract

Maternal morbidity and mortality rates in the United States have increased in the last two decades with a disproportionate impact on women of color. While numerous factors contribute to the inequities in pregnancy-related mortality, access to health insurance is among the most significant. Military Tricare models universal health care access; however, in studies looking at births in military treatment facilities, disparities still exist for women of color. This study analyzed maternal delivery outcomes for all women with Tricare coverage, including deliveries in the civilian sector. We analyzed data from 6.2 million births in the Centers for Disease Control (CDC) Wide-ranging Online Data for Epidemiology Research (WONDER) Linked Birth/Infant Death Records for 2017-2019. Data included all-cause morbidity (transfusions, perineal lacerations, uterine rupture, unplanned hysterectomy, and ICU admissions), severe maternal morbidity (SMM) excluding lacerations, and SMM excluding transfusion. Risk ratios were calculated by comparing overall maternal morbidity rates between Tricare, Medicaid, self-pay, and private insurance. In addition, risk ratios were calculated between insurance types stratified by race. In conclusion, there is an increased risk for women identifying as racial minorities for SMM and SMM excluding transfusion. While Tricare coverage seems to decrease the risk, the decrease is not significant and disparities in outcomes persist among women identifying as minorities. The risk of severe maternal morbidity remains elevated for women of color despite access to Tricare health insurance.

## Introduction

The phrase “maternal morbidity and mortality” (MMM) describes medical complications and deaths related to pregnancy and/or childbirth. Although global rates of MMM continue to improve, United States maternal mortality ratios (MMR) have worsened in the last two decades with a disproportionate effect on racial and ethnic minorities. The MMR is calculated by: MMR = (Number of maternal deaths / Number of live births) X 100,000. In other words, the MMR describes the risk of maternal death relative to the number of live births. The MMR quantifies the risk of death in a single pregnancy or a single live birth [1]. The American College of Obstetricians and Gynecologists (ACOG) reports that indigenous and Black women have a two to three times higher rate of maternal morbidity than their White counterparts [2]. Another group found that Black women have a 70% greater risk of severe maternal morbidity (SMM) than White women across all pregnancy intervals (antepartum, intrapartum, and postpartum) [3]. While numerous factors contribute to the inequities in pregnancy-related mortality, access to health insurance is among the most significant [4].

Tricare is the healthcare program for the Department of Defense serving active-duty members and their dependents, as well as retirees, their families, and survivors [5]. Tricare has several plan options to choose from, but all service members are enrolled in Tricare Prime, which is comparable to health maintenance organization (HMO) plans in the private sector. Active-duty service members have no cost sharing for approved healthcare services, and almost all care is delivered in military treatment facilities, but some care may be rendered in outside civilian settings. Most dependents of active-duty personnel are covered under Prime, but some choose Tricare Select, which is like a preferred provider organization (PPO) plan that features access to network and non-network providers.

Tricare coverage in the military closely models universal healthcare access. However, in the limited studies examining births in military treatment facilities, disparities in MMM were found for women of color [6,7]. The purpose of this study was to analyze maternal delivery outcomes for all women including service members, wives, and daughters of service members with any type of Tricare coverage. The deliveries analyzed took place at military treatment facilities or in civilian hospitals.

The data presented in this manuscript were previously presented as a podium presentation at the American College of Obstetricians and Gynecologists Armed Forces District Annual Meeting in October 2022 and as a poster presentation at the American College of Obstetricians and Gynecologists Annual Clinical Meeting in May of 2023.

## Materials and methods

Study design 

For this study, we analyzed retrospective data from 6.2 million births in the Centers for Disease Control (CDC) Wide-ranging Online Data for Epidemiology Research (WONDER) Linked Birth/Infant Death Records for 2017-2019. This is a freely searchable online database. Data included all-cause morbidity (transfusions, perineal lacerations, uterine rupture, unplanned hysterectomy, and ICU admissions), severe maternal morbidity (SMM) excluding lacerations, and SMM excluding transfusion. Data were stratified by insurance type into Tricare and Not Tricare. Not Tricare includes Medicaid, private insurance, and self-pay. Data were also stratified by maternal race (Black, White, Asian or Pacific Islander (API), American Indian or Alaska Native (AIAN), Native Hawaiian or Other Pacific Islander (NHOPI), and Other).

Inclusion and exclusion criteria

The CDC WONDER database that was used does not include deliveries following intrauterine fetal demise. Births associated with unknown payment type or maternal race were excluded from the analysis. Births occurring outside a hospital or before 20 weeks gestational age were also excluded.

Data collection

The WONDER Linked Infant Death database was used as the primary data source and includes infant, maternal, and paternal data for all live births in the US from 2017 to 2019. The database is freely searchable online. 

Statistical analysis

Rates of all-cause maternal morbidity (MM) were calculated along with the relative risk of MM for Tricare beneficiaries compared to all other payment methods. The CDC definition of SMM includes 21 unique ICD-10 codes for unexpected outcomes of childbirth but does not include perineal lacerations. There are also no distinguishing data in CDC WONDER regarding small- versus large-scale blood transfusions. With this in mind, we accounted for two categories of SMM: one excluding lacerations and another excluding transfusions. Statistical significance was set at alpha = 0.05. We used R (version 4.0; R Development Core Team, Vienna, Austria) statistical software and univariate analysis. When appropriate, we used either a t-test for continuous variables or chi-squared for categorical variables. We also calculated risk ratios.

## Results

The CDC WONDER database included data from 6,376,419 live births that occurred between 2017 and 2019. Our study analyzed 6,264,229 of these births. Of those cases analyzed, private insurance covered the majority of births at 51%, Medicaid covered the second largest group at 44%, followed by Tricare at ~2%, and those who were categorized as self-pay represented ~2.7% of the sample population (Table 1).

**Table 1:** Comparison of maternal morbidity by insurance coverage. Number of total births in the United States from 2017 to 2019 paid for by private insurance, Medicaid, Tricare, and self-pay methods, as well as the number of births complicated by all-cause maternal morbidity (MM), severe maternal morbidity (SMM) excluding perineal lacerations, and severe maternal morbidity excluding transfusions (SMM excl. Trans). Shown are risk ratios using private insurance as the comparison group with 95% confidence intervals.

		MM		SMM		SMM Excl. Trans	
Insurance	Births	N (%)	RR (95% CI)	N (%)	RR (95% CI)	N (%)	RR (95% CI)
Private	3,204,190	54,490 (1.70)	Control	19,984 (0.62)	Control	7,461 (0.23)	Control
Medicaid	2,767,308	34,053 (1.23)	0.83 (0.82 – 0.84)	21,160 (0.76)	1.11 (1.10 – 1.12)	7,965 (0.29)	1.12 (1.10 – 1.13)
Tricare	123,206	1,592 (1.29)	0.76 (0.73 – 0.80)	715 (0.58)	0.93 (0.87 – 1.00)	274 (0.22)	0.96 (0.85 – 1.08)
Self-Pay	169,525	2,398 (1.41)	0.84 (0.80 – 0.87)	1,276 (0.75)	1.20 (1.13 – 1.26)	541 (0.32)	1.35 (1.24 – 1.46)

Based on analysis of the data using a CI of 95%, the rate of MM was the lowest for the Medicaid group, followed closely by the Tricare group, with self-pay and private insurance demonstrating rates that appear slightly higher (p-value = 0.05). Compared to privately insured mothers, Tricare beneficiaries were at significantly decreased risk of MM. Similarly, rates of SMM and SMM excluding transfusion were lowest for the Tricare group; however, there was no statistically significant difference by insurance type for instances of SMM or SMM excluding transfusion among the different groups analyzed (Table 1).

**Figure 1:**
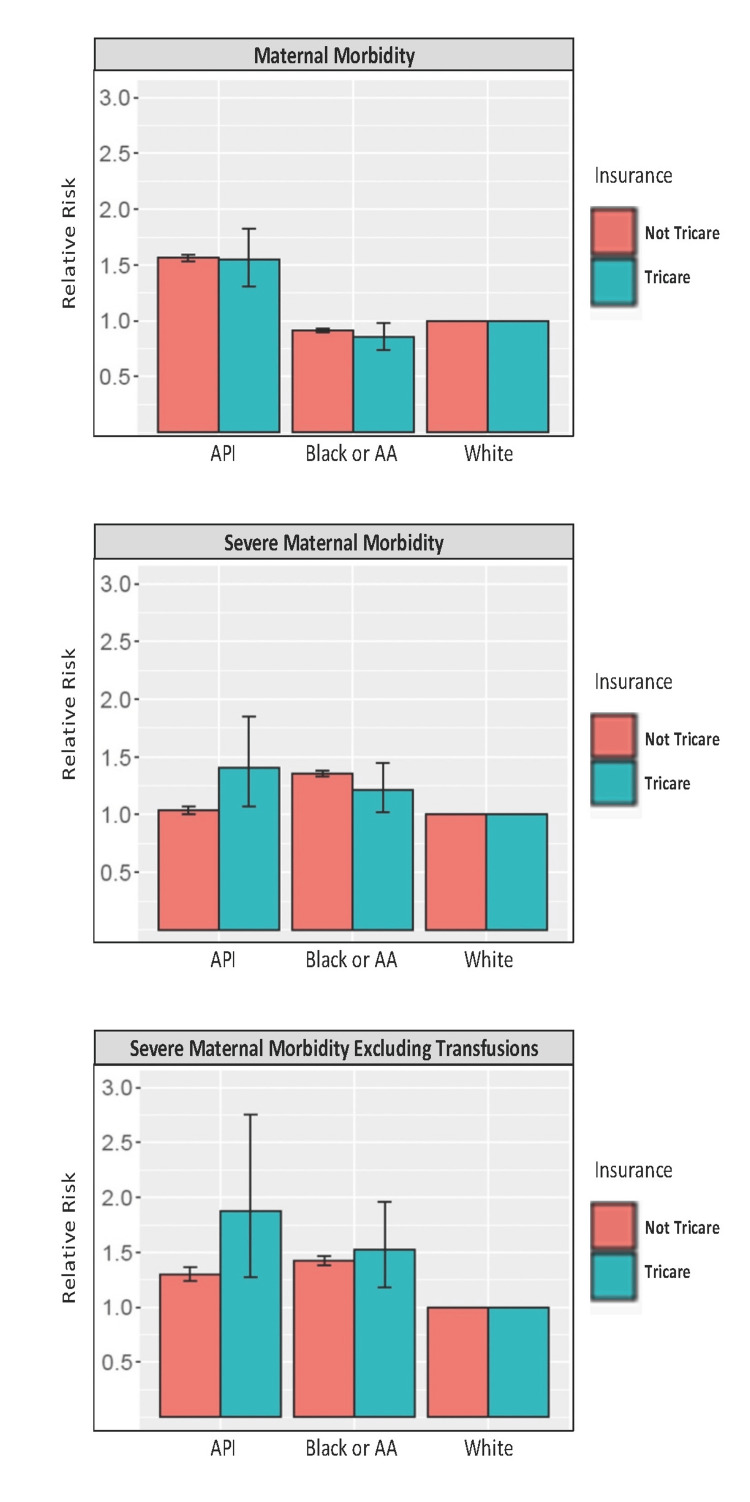
Maternal morbidity for Black and API women stratified by insurance coverage. Risk of maternal morbidity, severe maternal morbidity, and severe maternal morbidity excluding transfusions for minority identifying women (API or Black) as compared to White identifying women with 95% confidence intervals stratified by insurance coverage. Not Tricare insurance coverage includes Medicaid, private, and self-pay insurance. API = Asian Pacific Islander

The rates of MM were the lowest for mothers identifying as Black or more than one race. The rates of MM were the highest for mothers identifying as API. SMM was the lowest for White and API women and the highest for Black, AIAN, and NHOPI women. Similarly, the lowest rates of SMM excluding transfusion were for White women and the highest for AIAN, Black, and NHOPI women (Figure 1). 

When stratified by insurance status, Black women with Tricare were at a significantly lower risk for MM and significantly higher risk of SMM and SMM excluding transfusion compared to White women. The risk for Black women without Tricare is slightly greater for MM, SMM, and SMM excluding transfusion; however, these comparisons of Black women with and without Tricare fail to reach significance (Figure 1).

API women with Tricare coverage had significantly increased risk of MM, SMM, and SMM excluding transfusion. The risk for API women without Tricare appears to demonstrate a higher risk of MM, SMM, and SMM excluding transfusion. While the risk of SMM for both Tricare-covered and non-Tricare-covered API women is significantly increased, the difference in the risk is significantly greater for women with Tricare coverage (p-value = 0.05). There is not a significantly increased risk of MM or SMM without transfusion in comparing the two coverage groups for API women (Figure 1).

Compared to White women, Black women had a significantly decreased risk of MM, but a significantly increased risk of SMM and SMM excluding transfusion (p-value = 0.05). Risk of MM, SMM, and SMM excluding transfusion were all significantly increased for Asian women compared to White women (Table 2).87

**Table 2:** Complication risk by race. Number of total births in the United States from 2017-2019 by self-reported maternal race as well as number of births complicated by all-cause maternal morbidity (MM), severe maternal morbidity (SMM) which excludes perineal lacerations, and severe maternal morbidity excluding transfusions (SMM excl. Trans). Shown are risk ratios using White-identifying mothers as the comparison group with 95% confidence intervals. AIAN = American Indian and Alaskan Native, NHOPI = Native Hawaiian and Other Pacific Islander

		MM		SMM		SMM excl. Trans	
Race	Births	N (%)	RR (95% CI)	N (%)	RR (95% CI)	N (%)	RR (95% CI)
AIAN	70,783	1,259 (1.78)	1.25 (1.18 – 1.32)	920 (1.30)	2.03 (1.91 – 2.17)	247 (0.35)	1.50 (1.33 – 1.70)
Asian	497,094	11,678 (2.35)	1.56 (1.54 – 1.59)	3,290 (0.66)	1.04 (1.01 – 1.07)	1,545 (0.31)	1.30 (1.24 – 1.36)
Black and African American	902,621	11,571 (1.28)	0.91 (0.90 – 0.93)	8,326 (0.92)	1.36 (1.33 – 1.38)	3,234 (0.36)	1.42 (1.38 – 1.47)
More than one Race	198,289	2,406 (1.21)	0.85 (0.82 – 0.88)	1,352 (0.68)	1.07 (1.02 – 1.13)	533 (0.27)	1.16 (1.06 – 1.26
NHOPI	25,460	418 (1.64)	1.15 (1.05 – 1.27)	285 (1.12)	1.77 (1.57 – 1.99)	118 (0.46)	2.00 (1.67 – 2.39)
White	4,569,982	65,201 (1.43)	1.00 (control)	28,962 (0.63)	1.00 (control)	10,564 (0.23)	1.00 (control)

## Discussion

A systematic review of the literature regarding the social determinants of MMM in the US found that a lack of insurance coverage was associated with an increased incidence of maternal death and SMM compared to those covered publicly [8].

Fink et al. recently published an article in JAMA Network Open reviewing trends in MM and severe maternal morbidity during delivery-related hospitalizations in the US from 2008 to 2021. Interestingly, they found a trend towards improvement in the last 13 years but also noted worse outcomes for patients relying on Medicaid as their healthcare insurance [9]. 

A 2024 perspective article on strategies to reduce racial inequities in maternal health showed that insurance was a contributor. Those patients with Medicaid often lost care at six weeks postpartum, even if they had suffered significant medical comorbidity or complication in the pregnancy. They posed this lapse in healthcare or inadequate healthcare coverage as a contributor to the racial difference in SMM [10]. The military universal healthcare coverage continued coverage after the postpartum period ends without a lapse potentially improving the postpartum SMM that others with Medicaid face.

It has been shown that Medicaid expansion under the Affordable Care Act (ACA) was associated with significantly decreased rates of MM compared with non-expansion states, and these effects particularly benefitted non-Hispanic Black mothers [11]. That study suggests that Medicaid expansion could decrease racial disparities in MM and may be a primary target to lessen the SMM disparities that exist in the US.

However, racial and ethnic disparities still exist even when coverage is controlled for. One study examining complications among Medicaid beneficiaries with ectopic pregnancies showed that women from all racial and ethnic minority groups were significantly more likely than White women to experience complications [12]. Other studies have shown that racial and ethnic disparities in SMM persist even when risk adjusting for obstetric comorbidities [13].

Our study has several limitations. The CDC WONDER database is de-identified and aggregated, which does not allow us to assess confounding factors contributing to SMM. The parameters tracked in the database are gathered using birth certificates, which can be inaccurate and have varying formats from state to state. In addition, this study looks at outcomes based on payer status but does not delineate the location of delivery. Patients paying with Tricare could be delivered at a military treatment facility, a private hospital, or a university health system that also accepts Medicaid. 

Additionally, Black women appear to have a lower risk of MM for all insurance types compared to White women; however, this is likely attributed to perineal lacerations accounting for the largest proportion of MM. Notably, analysis of the sub-types of MM reported in CDC WONDER shows a concerning and significant increased risk of ICU admission for mothers identifying as racial minorities. While the CDC WONDER database does not delineate between all 21 ICD-10 codes for SMM, we hypothesize that women suffering from many of those 21 complications such as pulmonary embolism, sepsis, and heart failure would be accounted for within the ICU admission category.

## Conclusions

We speculate that universal coverage under Tricare would also have a mitigating effect on maternal morbidity disparities by race. We found partial support for that hypothesis. Tricare coverage appeared to decrease all-cause morbidity risk for Black women compared to White women, but the decrease was not statistically significant because all-cause MM for Black and White women was the same. API women also had higher rates of all-cause MM than White women. For our other two outcomes, SMM excluding laceration and SMM excluding transfusion, we identified a statistically significant increased risk for all women identifying as racial minorities compared to White women regardless of insurance type. Additionally, there is a significantly greater risk of SMM for API women who were Tricare beneficiaries.

Unfortunately, racial disparities in maternal morbidity and mortality persist, even with universal access to medical care in the military. In this study, we demonstrated how universal access to healthcare can mitigate several of these barriers to care; however, additional research is warranted to further investigate causal factors that could be driving these disparities for Tricare beneficiaries.

## References

[1] (2024). Maternal mortality ratio (per 100 000 live births). https://www.who.int/data/gho/indicator-metadata-registry/imr-details/26.

[2] (2024). Eliminating preventable maternal mortality and morbidity. https://www.acog.org/advocacy/policy-priorities/maternal-mortality-prevention.

[3] Liese KL, Mogos M, Abboud S, Decocker K, Koch AR, Geller SE (2019). Racial and ethnic disparities in severe maternal morbidity in the United States. J Racial Ethn Health Disparities.

[4] ACOG Committee Opinion (2024). Protecting and expanding Medicaid to improve women’s health. Obstet Gynecol.

[5] (2024). Health plans. https://www.tricare.mil/Plans/HealthPlans.

[6] Leonard SA, Main EK, Lyell DJ, Carmichael SL, Kennedy CJ, Johnson C, Mujahid MS (2022). Obstetric comorbidity scores and disparities in severe maternal morbidity across marginalized groups. Am J Obstet Gynecol MFM.

[7] Hamilton JL, Shumbusho D, Cooper D, Fletcher T, Aden J, Weir L, Keyser E (2021). Race matters: maternal morbidity in the military health system. Am J Obstet Gynecol.

[8] Wang E, Glazer KB, Howell EA, Janevic TM (2020). Social determinants of pregnancy-related mortality and morbidity in the United States: a systematic review. Obstet Gynecol.

[9] Fink DA, Kilday D, Cao Z (2023). Trends in maternal mortality and severe maternal morbidity during delivery-related hospitalizations in the United States, 2008 to 2021. JAMA Netw Open.

[10] Richardson MB, Toluhi AA, Baskin ML (2023). Community and systems contributors and strategies to reduce racial inequities in maternal health in the deep south: provider perspectives. Health Equity.

[11] Jarlenski M, Hutcheon JA, Bodnar LM, Simhan HN (2017). State Medicaid coverage of medically necessary abortions and severe maternal morbidity and maternal mortality. Obstet Gynecol.

[12] Eliason EL (2020). Adoption of Medicaid expansion is associated with lower maternal mortality. Womens Health Issues.

[13] Stulberg DB, Cain L, Dahlquist IH, Lauderdale DS (2016). Ectopic pregnancy morbidity and mortality in low-income women, 2004-2008. Hum Reprod.

